# *catena*-Poly[bis­[dimeth­yl(pyridine-κ*N*)indium(III)]-μ_4_-benzene-1,3-diolato-bis­[di­methyl­indium(III)]-μ_4_-benzene-1,3-diolato]

**DOI:** 10.1107/S1600536813028985

**Published:** 2013-10-26

**Authors:** Glen G. Briand, Andreas Decken, Marshall R. Hoey

**Affiliations:** a Department of Chemistry and Biochemistry, Mount Allison University, 63C York Street, Sackville, New Brunswick, E4L 1G8, Canada; b Department of Chemistry, University of New Brunswick, Fredericton, New Brunswick, E3B 5A3, Canada

## Abstract

The title compound, [In_2_(CH_3_)_4_(C_6_H_4_O_2_)(C_5_H_5_N)] or [{(CH_3_)_2_In}(1,3-O_2_C_6_H_4_){In(CH_3_)_2_(py)}]_*n*_, (py = pyridine) contains two crystallographically unique In^III^ ions which are in distorted tetra­hedral C_2_O_2_ and distorted trigonal-bipyramidal C_2_O_2_N coordination environments. The In^III^ coordination centers are bridged head-to-head *via* In—O bonds, yielding four-membered In_2_O_2_ rings and zigzag polymeric chains along [001].

## Related literature

For background to di­methyl­indium aryl­oxides, see: Briand *et al.* (2010 ▶); Beachley *et al.* (2003 ▶); Hausslein *et al.* (1999 ▶); Blake *et al.* (2011 ▶); Bradley *et al.* (1988 ▶); Trentler *et al.* (1997 ▶). For di­methyl­indium compounds with bidentate imine-alkoxide ligands, see: Hu *et al.* (1999 ▶); Wu *et al.* (1999 ▶); Pal *et al.* (2013 ▶); Lewinski *et al.* (2003 ▶); Ghoshal *et al.* (2007 ▶).
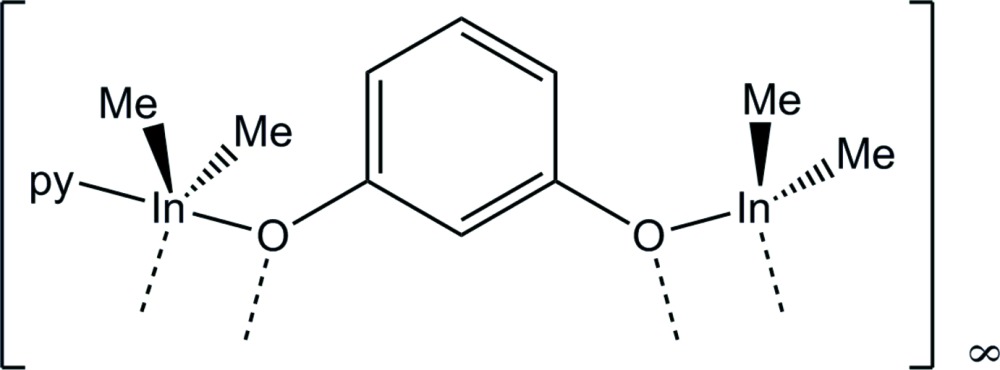


## Experimental

### 

#### Crystal data

[In_2_(CH_3_)_4_(C_6_H_4_O_2_)(C_5_H_5_N)]
*M* *_r_* = 476.97
Monoclinic, 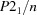

*a* = 9.1584 (17) Å
*b* = 14.075 (3) Å
*c* = 13.856 (3) Å
β = 90.106 (3)°
*V* = 1786.1 (6) Å^3^
*Z* = 4
Mo *K*α radiation
μ = 2.58 mm^−1^
*T* = 188 K
0.20 × 0.03 × 0.03 mm

#### Data collection

Bruker P4/SMART 1000 diffractometer
Absorption correction: multi-scan (*SADABS*; Sheldrick, 2008*a* ▶) *T*_min_ = 0.626, *T*_max_ = 0.938
12064 measured reflections
3967 independent reflections
2885 reflections with *I* > 2σ(*I*)
*R*_int_ = 0.039

#### Refinement

*R*[*F* ^2^ > 2σ(*F* ^2^)] = 0.035
*wR*(*F* ^2^) = 0.087
*S* = 1.16
3967 reflections
185 parameters
H-atom parameters constrained
Δρ_max_ = 1.02 e Å^−3^
Δρ_min_ = −0.72 e Å^−3^

### 

Data collection: *SMART* (Bruker, 1999 ▶); cell refinement: *SAINT* (Bruker, 2006 ▶); data reduction: *SAINT*; program(s) used to solve structure: *SHELXS97* (Sheldrick, 2008*b* ▶); program(s) used to refine structure: *SHELXL2013* (Sheldrick, 2008*b* ▶); molecular graphics: *DIAMOND* (Brandenburg, 2012 ▶); software used to prepare material for publication: *SHELXTL* (Sheldrick, 2008*b* ▶).

## Supplementary Material

Crystal structure: contains datablock(s) I. DOI: 10.1107/S1600536813028985/lh5663sup1.cif

Structure factors: contains datablock(s) I. DOI: 10.1107/S1600536813028985/lh5663Isup2.hkl

Supplementary material file. DOI: 10.1107/S1600536813028985/lh5663Isup3.cdx

Additional supplementary materials:  crystallographic information; 3D view; checkCIF report

## Figures and Tables

**Fig. 1.**
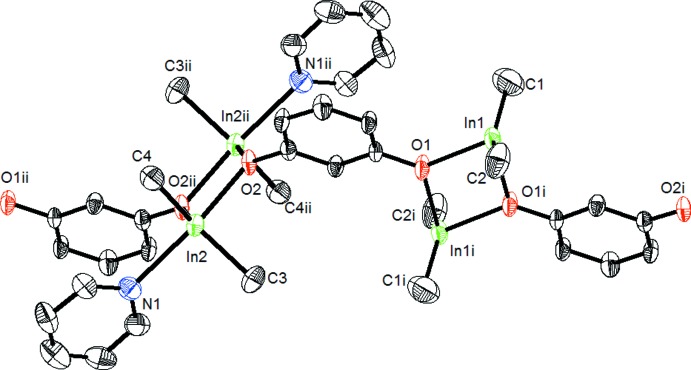
The molecular structure of (I), with displacement ellipsoids drawn at the 50% probability level. H atoms have been omitted for clarity. Symmetry transformations used to generate equivalent atoms: (i) -x, 1 - y, 1 - z; (ii) -x, 1 - y, 2 - z.

**Fig. 2.**
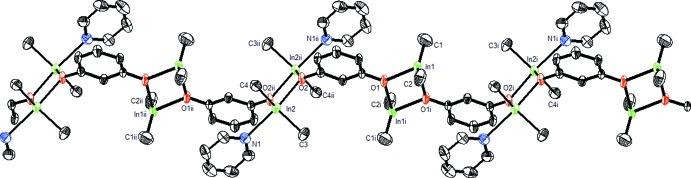
Part of the crystal structure of (I) showing the zigzag polymeric structure along [001], with displacement ellipsoids drawn at the 50% probability level. H atoms have been omitted for clarity. Symmetry transformations used to generate equivalent atoms: (i) -x, 1 - y, 1 - z; (ii) -x, 1 - y, 2 - z.

## supplementary crystallographic information

### 1. Comment

Dimethylindium aryloxides [Me_2_InOR]_2_ form dimeric structures in the solid state *via* intermolecular In—O coordinate bonding interactions (Briand *et al.*, 2010; Beachley *et al.*, 2003; Hausslein *et al.*, 1999; Blake *et al.*, 2011; Bradley *et al.*, 1988; Trentler *et al.*, 1997). These structures feature distorted tetrahedral geometries at In, distorted trigonal planar or slightly pyramidal geometries at O, and symmetric near planar In_2_O_2_ ring cores. Substitution of monodentate alkoxide (–OR) ligands with bidentate imine-alkoxide ligands additionally results in an intramolecular In—N coordination, yielding distorted trigonal bipyramidal In centres and asymmetric In_2_O_2_ rings (Hu *et al.*, 1999; Wu *et al.*, 1999; Pal *et al.*, 2013; Lewinski *et al.*, 2003; Ghoshal *et al.*, 2007). The molecular structure of (I) (Fig. 1) shows two crystallographically unique In atoms. In addition to the In1—O1 bond, In1 exhibits an intermolecular In1—O1^i^ interaction. This results in a distorted tetrahedral C_2_O_2_ bonding environment for indium [C1—In1—C2 = 144.3 (3), O1—In1—O1^i^ = 73.87 (14)°] and a symmetric In_2_O_2_ ring structure [In1—O1 = 2.172 (3), In1—O1^i^ = 2.174 (3) Å]. Similarly, In2 is coordinated to two methyl C atoms [C3 and C4] and two aryloxide O atoms [O2 and O2^ii^], but is is also coordinated by the N atom of a pyridine molecule [2.486 (4) Å]. This results in a distorted trigonal bypyramidal C_2_O_2_N bonding environment for In, with the two methyl C atoms and a bridging O atom in equatorial positions [C3—In2—C4 = 142.5 (2), C3—In2—O2^ii^ = 107.8 (2), C4—In2—O2^ii^ = 109.25 (19)°], and the pyridine N atom and a bridging O atom in axial positions [O2—In2—N1 = 156.63 (13)°]. The axial In2—O2 bond distance [2.330 (3) Å] is longer than the equatorial In2—O2^ii^ bond distance [2.152 (3) Å], presumably as a result of the *trans* influence of the pyridine N atom, resulting in an asymmetric In_2_O_2_ ring. The two unique In_2_O_2_ rings are bridged by the 1,3-benzenediolate phenyl ring, giving a zigzag polymeric structure along [001] (Fig. 2).

### 2. Experimental

Under an atmosphere of dinitrogen, InMe_3_ (0.250 g, 1.56 mmol) was dissolved in diethyl ether (10 ml). Pyridine (0.125 g, 1.56 mmol) was added and the solution stirred for 30 min. Resorcinol (0.088 g, 0.78 mmol) was then added and the reaction mixture stirred for an additional 1 h. The reaction was then filtered and the filtrate allowed to sit at 296K. After 1 d, the solution was filtered to yield colourless crystals of [Me_2_In(1,3-O_2_C_6_H_4_)InMe_2_(py)]_∞_ (0.122 g, 0.256 mmol, 33%). Anal. Calc. for C_15_H_21_In_2_NO_2_: C, 37.77; H, 4.44; N, 2.94. Found: C, 38.32; H, 4.47; N, 2.88. F T—IR (ATR, cm^-1^): 618 w, 698 s, 748 m, 772 w, 829 w, 844 w, 968 s, 1003 w, 1034 w, 1142 s, 1171 s, 1213 w, 1249 m, 1299 m, 1427 w, 1441 w, 1482 m, 1467 m, 1573 s, 2283 w, 2475 w, 2881 m, 3004 m. FT-Raman (cm^-1^): 487 vs [ν*_sym_* (Me—In—Me)], 524 w [ν*_asym_* (Me—In—Me)], 994 m, 1034 m, 1159 m, 1305 w, 1586 w, 2920 m, 2982 w, 3064 m.

### 3. Refinement

Hydrogen atoms were included in calculated positions and refined using a riding model.

### Crystal data

**Table:**

[In_2_(CH_3_)_4_(C_6_H_4_O_2_)(C_5_H_5_N)]	*F*(000) = 928
*M**_r_* = 476.97	*D*_x_ = 1.774 Mg m^−^^3^
Monoclinic, *P*2_1_/*n*	Mo *K*α radiation, λ = 0.71073 Å
Hall symbol: -P 2yn	Cell parameters from 5671 reflections
*a* = 9.1584 (17) Å	θ = 2.7–27.9°
*b* = 14.075 (3) Å	µ = 2.58 mm^−^^1^
*c* = 13.856 (3) Å	*T* = 188 K
β = 90.106 (3)°	Rod, colourless
*V* = 1786.1 (6) Å^3^	0.20 × 0.03 × 0.03 mm
*Z* = 4	

### Data collection

**Table:**

Bruker P4/SMART 1000 diffractometer	3967 independent reflections
Radiation source: fine-focus sealed tube, K760	2885 reflections with *I* > 2σ(*I*)
Graphite monochromator	*R*_int_ = 0.039
φ and ω scans	θ_max_ = 27.5°, θ_min_ = 2.1°
Absorption correction: multi-scan (*SADABS*; Sheldrick, 2008*a*)	*h* = −10→11
*T*_min_ = 0.626, *T*_max_ = 0.938	*k* = −18→18
12064 measured reflections	*l* = −16→17

### Refinement

**Table:**

Refinement on *F*^2^	Primary atom site location: structure-invariant direct methods
Least-squares matrix: full	Secondary atom site location: difference Fourier map
*R*[*F*^2^ > 2σ(*F*^2^)] = 0.035	Hydrogen site location: inferred from neighbouring sites
*wR*(*F*^2^) = 0.087	H-atom parameters constrained
*S* = 1.16	*w* = 1/[σ^2^(*F*_o_^2^) + (0.0339*P*)^2^ + 1.2232*P*] where *P* = (*F*_o_^2^ + 2*F*_c_^2^)/3
3967 reflections	(Δ/σ)_max_ = 0.001
185 parameters	Δρ_max_ = 1.02 e Å^−^^3^
0 restraints	Δρ_min_ = −0.72 e Å^−^^3^

### Special details

**Table:**

Experimental. Crystal decay was monitored by repeating the initial 50 frames at the end of the data collection and analyzing duplicate reflections.
Geometry. All e.s.d.'s (except the e.s.d. in the dihedral angle between two l.s. planes) are estimated using the full covariance matrix. The cell e.s.d.'s are taken into account individually in the estimation of e.s.d.'s in distances, angles and torsion angles; correlations between e.s.d.'s in cell parameters are only used when they are defined by crystal symmetry. An approximate (isotropic) treatment of cell e.s.d.'s is used for estimating e.s.d.'s involving l.s. planes.
Refinement. Reflections were merged by *SHELXL* according to the crystal class for the calculation of statistics and refinement._reflns_Friedel_fraction is defined as the number of unique Friedel pairs measured divided by the number that would be possible theoretically, ignoring centric projections and systematic absences.

### Fractional atomic coordinates and isotropic or equivalent isotropic displacement parameters (Å^2^)

**Table:**

	*x*	*y*	*z*	*U*_iso_*/*U*_eq_	
In1	0.12136 (4)	0.57110 (3)	0.43640 (3)	0.03464 (12)	
In2	0.11428 (4)	0.39665 (3)	1.01934 (3)	0.02955 (11)	
O1	0.0623 (4)	0.5253 (3)	0.5809 (2)	0.0361 (8)	
O2	0.0754 (3)	0.5316 (2)	0.9254 (2)	0.0300 (8)	
N1	0.0635 (5)	0.2789 (3)	1.1488 (3)	0.0396 (11)	
C1	0.0184 (8)	0.7054 (5)	0.4259 (6)	0.073 (2)	
H1A	0.0580	0.7480	0.4755	0.109*	
H1B	−0.0870	0.6980	0.4356	0.109*	
H1C	0.0365	0.7325	0.3619	0.109*	
C2	0.3108 (7)	0.4920 (5)	0.3979 (5)	0.0631 (19)	
H2A	0.3153	0.4856	0.3276	0.095*	
H2B	0.3060	0.4288	0.4275	0.095*	
H2C	0.3981	0.5253	0.4211	0.095*	
C3	0.0431 (7)	0.3018 (4)	0.9068 (4)	0.0562 (17)	
H3A	0.1234	0.2910	0.8616	0.084*	
H3B	−0.0397	0.3302	0.8724	0.084*	
H3C	0.0130	0.2412	0.9353	0.084*	
C4	0.3053 (6)	0.4553 (4)	1.0846 (4)	0.0454 (15)	
H4A	0.3067	0.5242	1.0746	0.068*	
H4B	0.3923	0.4269	1.0553	0.068*	
H4C	0.3047	0.4417	1.1540	0.068*	
C5	0.1383 (5)	0.5416 (3)	0.6650 (3)	0.0270 (10)	
C6	0.0693 (5)	0.5259 (3)	0.7530 (3)	0.0277 (11)	
H6	−0.0282	0.5029	0.7544	0.033*	
C7	0.1435 (5)	0.5439 (3)	0.8395 (3)	0.0264 (10)	
C8	0.2872 (5)	0.5754 (4)	0.8361 (4)	0.0314 (11)	
H8	0.3395	0.5870	0.8941	0.038*	
C9	0.3536 (6)	0.5897 (4)	0.7479 (4)	0.0371 (13)	
H9	0.4518	0.6115	0.7462	0.044*	
C10	0.2808 (5)	0.5731 (3)	0.6622 (3)	0.0311 (11)	
H10	0.3282	0.5832	0.6022	0.037*	
C11	−0.0627 (6)	0.2311 (4)	1.1471 (5)	0.0464 (14)	
H11	−0.1250	0.2380	1.0927	0.056*	
C12	−0.1062 (7)	0.1719 (4)	1.2216 (5)	0.0572 (17)	
H12	−0.1960	0.1383	1.2176	0.069*	
C13	−0.0192 (8)	0.1624 (5)	1.3002 (5)	0.066 (2)	
H13	−0.0476	0.1223	1.3520	0.080*	
C14	0.1099 (8)	0.2112 (5)	1.3044 (5)	0.0614 (18)	
H14	0.1723	0.2061	1.3590	0.074*	
C15	0.1467 (7)	0.2677 (4)	1.2274 (4)	0.0479 (15)	
H15	0.2369	0.3010	1.2302	0.057*	

### Atomic displacement parameters (Å^2^)

**Table:**

	*U*^11^	*U*^22^	*U*^33^	*U*^12^	*U*^13^	*U*^23^
In1	0.0337 (2)	0.0504 (2)	0.0198 (2)	−0.00910 (16)	0.00038 (15)	0.00502 (16)
In2	0.0284 (2)	0.0370 (2)	0.02328 (19)	0.00222 (15)	0.00004 (14)	−0.00206 (15)
O1	0.035 (2)	0.058 (2)	0.0152 (17)	−0.0138 (17)	−0.0001 (15)	0.0008 (16)
O2	0.0281 (18)	0.047 (2)	0.0149 (17)	0.0054 (15)	0.0021 (14)	0.0016 (15)
N1	0.039 (3)	0.040 (3)	0.040 (3)	0.001 (2)	0.004 (2)	0.003 (2)
C1	0.080 (5)	0.058 (4)	0.081 (5)	−0.005 (4)	−0.005 (4)	0.018 (4)
C2	0.043 (4)	0.103 (6)	0.044 (4)	−0.001 (4)	0.004 (3)	−0.014 (4)
C3	0.078 (5)	0.054 (4)	0.037 (3)	−0.002 (3)	0.002 (3)	−0.018 (3)
C4	0.034 (3)	0.051 (4)	0.051 (4)	−0.007 (3)	−0.011 (3)	0.011 (3)
C5	0.035 (3)	0.032 (3)	0.014 (2)	−0.001 (2)	−0.001 (2)	0.0007 (19)
C6	0.022 (2)	0.040 (3)	0.022 (3)	−0.004 (2)	−0.001 (2)	0.000 (2)
C7	0.031 (3)	0.030 (3)	0.019 (2)	0.006 (2)	0.001 (2)	0.001 (2)
C8	0.028 (3)	0.048 (3)	0.018 (2)	−0.003 (2)	−0.004 (2)	−0.001 (2)
C9	0.025 (3)	0.058 (4)	0.029 (3)	−0.007 (2)	−0.001 (2)	0.004 (2)
C10	0.031 (3)	0.046 (3)	0.016 (2)	−0.007 (2)	0.003 (2)	−0.001 (2)
C11	0.049 (4)	0.040 (3)	0.050 (4)	−0.003 (3)	−0.002 (3)	0.001 (3)
C12	0.049 (4)	0.047 (4)	0.076 (5)	−0.010 (3)	0.009 (4)	0.009 (3)
C13	0.065 (5)	0.065 (5)	0.069 (5)	0.001 (4)	0.013 (4)	0.024 (4)
C14	0.073 (5)	0.069 (5)	0.042 (4)	0.011 (4)	−0.004 (3)	0.020 (3)
C15	0.050 (4)	0.048 (4)	0.046 (4)	0.001 (3)	0.002 (3)	0.008 (3)

### Geometric parameters (Å, º)

**Table:**

In1—C1	2.118 (7)	C3—H3C	0.9800
In1—C2	2.130 (6)	C4—H4A	0.9800
In1—O1	2.172 (3)	C4—H4B	0.9800
In1—O1^i^	2.174 (3)	C4—H4C	0.9800
In2—C4	2.134 (5)	C5—C10	1.379 (7)
In2—O2^ii^	2.152 (3)	C5—C6	1.393 (6)
In2—C3	2.152 (5)	C6—C7	1.400 (6)
In2—O2	2.330 (3)	C6—H6	0.9500
In2—N1	2.486 (4)	C7—C8	1.390 (7)
O1—C5	1.376 (5)	C8—C9	1.380 (7)
O1—In1^i^	2.174 (3)	C8—H8	0.9500
O2—C7	1.355 (5)	C9—C10	1.381 (7)
O2—In2^ii^	2.152 (3)	C9—H9	0.9500
N1—C11	1.337 (7)	C10—H10	0.9500
N1—C15	1.338 (7)	C11—C12	1.385 (8)
C1—H1A	0.9800	C11—H11	0.9500
C1—H1B	0.9800	C12—C13	1.355 (9)
C1—H1C	0.9800	C12—H12	0.9500
C2—H2A	0.9800	C13—C14	1.368 (9)
C2—H2B	0.9800	C13—H13	0.9500
C2—H2C	0.9800	C14—C15	1.373 (8)
C3—H3A	0.9800	C14—H14	0.9500
C3—H3B	0.9800	C15—H15	0.9500
			
C1—In1—C2	144.3 (3)	H3A—C3—H3C	109.5
C1—In1—O1	102.5 (2)	H3B—C3—H3C	109.5
C2—In1—O1	106.3 (2)	In2—C4—H4A	109.5
C1—In1—O1^i^	101.9 (2)	In2—C4—H4B	109.5
C2—In1—O1^i^	106.1 (2)	H4A—C4—H4B	109.5
O1—In1—O1^i^	73.87 (14)	In2—C4—H4C	109.5
C4—In2—O2^ii^	109.25 (19)	H4A—C4—H4C	109.5
C4—In2—C3	142.5 (2)	H4B—C4—H4C	109.5
O2^ii^—In2—C3	107.8 (2)	O1—C5—C10	120.5 (4)
C4—In2—O2	92.62 (17)	O1—C5—C6	119.0 (4)
O2^ii^—In2—O2	72.15 (13)	C10—C5—C6	120.4 (4)
C3—In2—O2	93.2 (2)	C5—C6—C7	120.0 (4)
C4—In2—N1	96.11 (19)	C5—C6—H6	120.0
O2^ii^—In2—N1	84.49 (13)	C7—C6—H6	120.0
C3—In2—N1	93.0 (2)	O2—C7—C8	120.5 (4)
O2—In2—N1	156.63 (13)	O2—C7—C6	120.3 (4)
C5—O1—In1	127.2 (3)	C8—C7—C6	119.1 (4)
C5—O1—In1^i^	126.0 (3)	C9—C8—C7	119.8 (5)
In1—O1—In1^i^	106.13 (14)	C9—C8—H8	120.1
C7—O2—In2^ii^	128.7 (3)	C7—C8—H8	120.1
C7—O2—In2	121.6 (3)	C8—C9—C10	121.5 (5)
In2^ii^—O2—In2	107.85 (13)	C8—C9—H9	119.2
C11—N1—C15	116.4 (5)	C10—C9—H9	119.2
C11—N1—In2	119.1 (4)	C5—C10—C9	119.1 (4)
C15—N1—In2	124.0 (4)	C5—C10—H10	120.4
In1—C1—H1A	109.5	C9—C10—H10	120.4
In1—C1—H1B	109.5	N1—C11—C12	122.7 (6)
H1A—C1—H1B	109.5	N1—C11—H11	118.7
In1—C1—H1C	109.5	C12—C11—H11	118.7
H1A—C1—H1C	109.5	C13—C12—C11	119.2 (6)
H1B—C1—H1C	109.5	C13—C12—H12	120.4
In1—C2—H2A	109.5	C11—C12—H12	120.4
In1—C2—H2B	109.5	C12—C13—C14	119.5 (6)
H2A—C2—H2B	109.5	C12—C13—H13	120.3
In1—C2—H2C	109.5	C14—C13—H13	120.3
H2A—C2—H2C	109.5	C13—C14—C15	118.1 (7)
H2B—C2—H2C	109.5	C13—C14—H14	120.9
In2—C3—H3A	109.5	C15—C14—H14	120.9
In2—C3—H3B	109.5	N1—C15—C14	124.1 (6)
H3A—C3—H3B	109.5	N1—C15—H15	118.0
In2—C3—H3C	109.5	C14—C15—H15	118.0

Symmetry codes: (i) −*x*, −*y*+1, −*z*+1; (ii) −*x*, −*y*+1, −*z*+2.

## References

[bb1] Beachley, O. T. Jr, MacRae, D. J. & Kovalevsky, A. Y. (2003). *Organometallics*, **22**, 1690–1695.

[bb2] Blake, M. P., Schwarz, A. D. & Mountford, P. (2011). *Organometallics*, **30**, 1202–1214.

[bb3] Bradley, D. C., Frigo, D. M., Hursthouse, M. B. & Hussain, B. (1988). *Organometallics*, **7**, 1112–1115.

[bb4] Brandenburg, K. (2012). *DIAMOND* Crystal Impact GbR, Bonn, Germany.

[bb5] Briand, G. G., Decken, A. & Hamilton, N. S. (2010). *Dalton Trans.* **39**, 3833–3841. 10.1039/b927128g 20372707

[bb6] Bruker (1999). *SMART* Bruker AXS Inc., Madison, Wisconsin, USA.

[bb7] Bruker (2006). *SAINT* Bruker AXS Inc., Madison, Wisconsin, USA.

[bb8] Ghoshal, S., Wadawale, A., Jain, V. K. & Nethaji, M. (2007). *J. Chem. Res.* pp. 221–225.

[bb9] Hausslein, M., Hausen, H.-D., Klinkhammer, K. W., Weidlein, J. & Merz, K. (1999). *Z. Anorg. Allg. Chem.* **625**, 1608–1618.

[bb10] Hu, J.-Z., Yang, M., Wu, X.-S., Pan, Y., Liu, Y.-J. & Sun, X.-Z. (1999). *Wuji Huaxue Xuebao (Chin.) (Chin. J. Inorg. Chem.)*, **15**, 347–350.

[bb11] Lewinski, J., Zachara, J., Starowieyski, K. B., Ochal, Z., Justyniak, I., Kopec, T., Stolarzewicz, P. & Dranka, M. (2003). *Organometallics*, **22**, 3773–3780.

[bb12] Pal, M. K., Kushwah, N., Manna, D., Wadawale, A., Sudarsan, V., Ghanty, T. K. & Jain, V. K. (2013). *Organometallics*, **32**, 104–111.

[bb13] Sheldrick, G. M. (2008*a*). *SADABS* University of Göttingen, Germany.

[bb14] Sheldrick, G. M. (2008*b*). *Acta Cryst.* A**64**, 112–122. 10.1107/S0108767307043930 18156677

[bb15] Trentler, T. J., Goel, S. C., Hickman, K. M., Viano, A. M., Chiang, M. Y., Beatty, A. M., Gibbons, P. C. & Buhro, W. E. (1997). *J. Am. Chem. Soc.* **119**, 2172–2181.

[bb16] Wu, X.-S., Pan, Y., Sun, X.-Z. & Zhu, Y. (1999). *Jiegou Huaxue (Chin. J. Struct. Chem.)*, **18**, 418–422.

